# Ethnicity and gender related differences in extended intraesophageal pH monitoring parameters in infants: a retrospective study

**DOI:** 10.1186/1471-2431-5-24

**Published:** 2005-07-18

**Authors:** Dena Nazer, Ronald Thomas, Vasundhara Tolia

**Affiliations:** 1 Children's Hospital of Michigan, Carman and Ann Adams Department of Pediatrics, 3901 Beaubien Street, Detroit, MI 48201, USA; 2 Children's Research Center of Michigan, 3901 Beaubien Street, Detroit, MI 48201, USA

## Abstract

**Background:**

Gastroesophageal reflux disease (GERD) is believed to be more common in adult males as compared to females. It also has been shown in adults to be more common in Caucasians. We wanted to determine ethnicity and gender related differences for extended pH monitoring parameters in infancy.

**Methods:**

Extended pH monitoring data (EPM) from infants <1 year of age were reviewed. Results were classified in two groups, as control and Gastroesophageal reflux disease (GERD) group based on the reflux index (RI). The GERD group had RI of equal to or more than 5% of total monitoring period. The parameters of RI, total number of episodes of pH < 4, and the number of episodes with pH < 4 lasting more than 5 minutes were compared by genders and by ethnic groups, Caucasians and African American (AA).

**Results:**

There were 569 infants, 388 controls, 181 with GERD (320 males, 249 females; 165 Caucasians, 375 AA). No statistical difference in EPM parameters was detected between genders in both groups. However, Caucasian infants had a significantly higher incidence of GERD than AA infants (p = 0.036). On stratifying by gender, Caucasian females had a significantly higher number of reflux episodes >5 minutes as compared to AA females in the control group (p = 0.05). Furthermore, Caucasian females with GERD showed an overall higher trend for all parameters. Caucasian males had a trend for higher mean number of reflux episodes as compared to AA males in the control group (p = 0.09).

**Conclusion:**

Although gender specific control data do not appear warranted in infants undergoing EPM, ethnic differences related to an overall increased incidence of pathologic GERD in Caucasian infants should be noted.

## Background

Gastroesophageal reflux (GER) in infancy is a frequent cause for referral to a pediatric gastroenterologist [1]. It is considered physiologic if the infant is thriving and suffers no complications of reflux [1]. Pathologic gastroesophageal reflux disease (GERD) is associated with malnutrition, respiratory disorders, esophagitis, or other complications [1]. Although history and physical examination suffice to diagnose it empirically, further diagnostic evaluation is needed in certain cases for confirming the diagnosis and assessing its severity.

Extended pH monitoring (EPM) has been used in the diagnosis of GERD due to its high sensitivity and specificity [2]. While control data are available for classifying GER as being physiologic or pathologic for different ages in infancy, gender and ethnicity related differences have not been previously evaluated [3,4]. Extended pH monitoring data from adults have shown that males have significantly more physiologic and pathologic GER as compared to females [5-7]. Overall incidence of reflux disease and its complications such as Barrett's esophagus is higher in Caucasian males [8]. Gender related differences exist in other physiologic and disease states as early as neonatal age [9]. It is therefore important to assess if such differences exist in reflux parameters as well. We performed a retrospective study to determine if there were any quantitative differences in the EPM parameters between male and female infants being evaluated for GER and to assess ethnicity related differences in infancy.

## Methods

We reviewed pH-monitoring data on infants <1 year of age who underwent EPM between the periods from Jan 1^st^, 1995 to December 31^st^, 1998. Presenting symptoms in the referred infants included gastrointestinal symptoms (vomiting, gagging, or nasopharyngeal reflux), respiratory symptoms (choking, coughing, wheezing, or acute life threatening events), and other nonspecific symptoms (irritability, or failure to thrive). The indications and decision to perform an EPM were made by our Gastroenterology specialists.

After parental consent, all patients underwent an 18–24 hour EPM study as inpatients after evaluation by a gastroenterologist. The parents were encouraged to continue their routine feedings and activities to represent the normal variations in esophageal pH values as best as possible. EPM was performed using a portable pH recorder (Digitrapper, MKIII, Synectics Medical, Inc., Irving, TX). A flexible, disposable probe with a 1.6 mm outer diameter with a built-in internal reference electrode (Zinetics medical, Salt lake city, UT) was passed nasally into the fasted stomach after calibrating it at pH 1.0 and 7.0 before each study. The probe was then withdrawn to 87% of the distance from the nares to the lower esophageal sphincter as described by Strobel et al. [10]. Patients were fed formula or asked to continue nursing to maintain feeding regimen as at home during the study. All patients were kept off their home medications, specifically proton pump inhibitors, H2 receptor antagonists, prokinetics, and antacids, for at least 72 hours prior to the study. Event markers were used to indicate the beginning and end of feeding, regurgitation, coughing and choking. These events were mostly recorded by the parents and occasionally by the nursing staff. Meal periods were not excluded form the analysis.

Esophogram software from Synectics (Irving, TX) was used to analyze the data. Total percentage of time pH was <4.0 (reflux index), total number of episodes of reflux and number of episodes lasting >5 minutes were evaluated. Gastroesophageal reflux disease (GERD) was diagnosed if reflux index was ≥5%. Based on reflux index results, patients were divided into two groups, those with normal EPM as control or physiologic group versus GERD group with abnormal esophageal pH exposure.

### Data analysis

EPM parameters, continuously scaled, were compared between males and females using a parametric independent samples t-test. Differences in pathological and physiological outcome between ethnicity groups were examined using a Fisher's Exact Chi-square test. Statistical significance was set at a p-value ≤ 0.05, 2-tailed. Analyses on EPM parameters on subsets of gender and ethnicity were conducted using a parametric Two-Factor Analysis-of-Variance. All analyses were performed using Statistical Package for Social Sciences (SPSS), Version 11.5.

## Results

The extended distal esophageal pH monitoring was performed in 569 infants under a year of age during the review period. There were 320 males and 249 females. The mean age of patients was 3.93 ± 2.57 months (3.90 ± 2.45 months for males and 3.98 ± 2.71 months for females).

The majority of infants had more than one presenting symptom. Specifically, of 495 infants, (87%) had at least one gastrointestinal symptom; respiratory symptoms were noted in 307 patients (54%) and nonspecific symptoms were found in 151 patients (26.6%).

Physiologic amount of reflux was identified in 388 subjects, considered control group. Of these 219 (56.4%) were males and 169 (43.6%) were females. Pathologic GERD was present in 181 patients i.e., GERD group, [101 (55.8%) males and 80 (44.2%) females]. The mean age was 3.83 ± 2.53 months for the control group and 4.17 ± 2.63 months for the GERD group. The distribution of gender in all groups was similar.

Tables 1 and 2 show comparison of EPM parameters between the two genders in the control and GERD group respectively. There was no statistical difference between the two genders regarding any of the EPM parameters in either group. When ethnic distribution was compared, the control group had 265 AA and 101 Caucasian infants. The remaining 22 were of other ethnic origins (Middle Eastern, Hispanic, and Orientals). In the GERD group, 110 infants were AA, 64 were Caucasian and the remaining 7 were of other ethnicity (Middle Eastern and Hispanic). Since the numbers for other ethnic groups are low, only the two major races were compared. The mean age in both groups for both races was similar (4.01 ± 2.54 months for AA and 3.90 ± 2.66 months for Caucasians).

**Table 1:** Patients with normal EPM findings (control group) as divided by gender

	Males	Females	p value
Number of patients	219	169	
Mean age (months) ± SD	3.81 ± 2.58	3.85 ± 2.47	0.90
Mean reflux index % ± SD	2.11 ± 1.51	2.20 ± 1.52	0.57
Mean number of reflux episodes ± SD	45.72 ± 37.04	46.84 ± 36.80	0.77
Mean number of reflux episodes >5 minutes	0.62 ± 0.93	0.66 ± 0.96	0.67

SD: standard deviation

**Table 2:** Patients with abnormal EPM findings (GERD group) as divided by gender

	Males	Females	p value
Number of patients	101	80	NS
Mean age (months ± SD)	4.08 ± 2.16	4.28 ± 3.14	0.62
Mean reflux index % ± SD	11.47 ± 7.90	12.45 ± 9.32	0.44
Mean number of reflux episodes ± SD	102.59 ± 61.5	107.55 ± 91.81	0.66
Mean number of reflux episodes >5 minutes	3.34 ± 2.75	4.03 ± 4.59	0.21

SD: standard deviation

**Figure 1:**
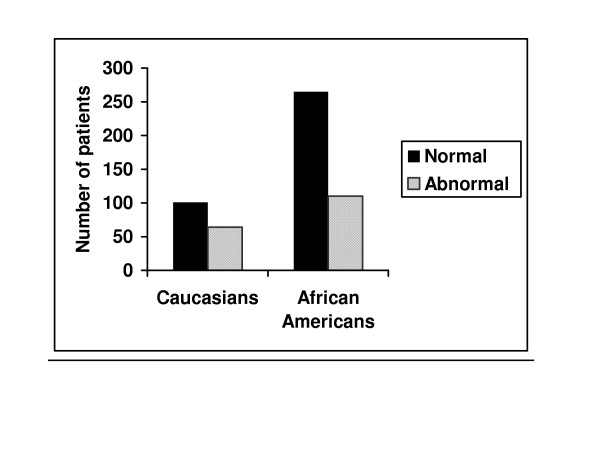
Shows higher incidence of GERD in Caucasians as compared to AA (P = 0.036).

More Caucasian infants (38.8%) had GERD compared to AA infants (29.3%) suggesting a higher prevalence of GERD in Caucasian infants (p = 0.036) (figure 1). In addition, Caucasian infants generally had higher values for all EPM parameters and higher trend for the total number of reflux episodes and reflux episodes >5 minutes in duration in the GERD group as compared to AA (tables 3 and 4).

**Table 3:** Patients with normal EPM findings (control group) as divided by ethnicity

	Caucasians	African Americans	p
Number of patients	101	265	
Mean age (months) ± SE	3.73 ± 0.26	3.91 ± 0.16	0.56
Mean reflux index % ± SE	2.38 ± 0.50	2.12 ± 0.31	0.65
Mean number of reflux episodes ± SE	54.39 ± 5.30	44.6 ± 3.27	0.12
Mean number of reflux episodes >5 minutes ± SE	0.78 ± 0.22	0.61 ± 0.14	0.52

SE: standard error

**Table 4:** Patients with abnormal EPM findings (GERD group) as divided by ethnicity

	Caucasians	African Americans	p
Number of patients	64	110	
Mean age(months) ± SE	4.17 ± 0.32	4.24 ± 0.24	0.86
Mean reflux index % ± SE	12.19 ± 0.63	11.97 ± 0.48	0.79
Mean number of reflux episodes ± SE	115.27 ± 6.66	100.68 ± 5.08	0.08
Mean number of reflux episodes >5 minutes ± SE	4.06 ± 0.28	3.40 ± 0.21	0.06

SE: standard error

We further stratified data based on ethnicity and gender. Caucasian females in the GERD group had an overall trend for higher values of all EPM parameters as compared to Caucasian males, AA males and AA females. They were also significantly older than Caucasian males (p = 0.03). Moreover, Caucasian females showed a significantly higher number of reflux episodes greater than 5 minutes in duration when compared to AA females in the control group (p = 0.05). The Caucasian males from the control group also had a trend for higher mean number of reflux episodes in comparison to AA male cohort (p = 0.09).

## Discussion

Gastroesophageal reflux (GER) is a common disorder in infancy [1,11]. The rate of GER diagnosis has increased more than 20 fold in hospitalized infants under a year of age over the past few decades [12]. Whether this increase in diagnosis is due to a true increase in pathologic reflux or an increased awareness of diagnosis remains undetermined.

EPM is considered a reliable method of measuring acid reflux [13]. It establishes the presence of abnormal acid reflux, determines if there is a temporal association between acid reflux and frequently occurring symptoms, and assesses the adequacy of therapy in patients who do not respond to treatment with acid suppressants [13]. Although considered the gold standard of diagnosing GERD previously [14], EPM currently should be viewed with limitations [1,2]. EPM may be of normal range in some patients, but brief episodes of GER may cause complications such as ALTE, cough, or aspiration pneumonia [13].

The prevalence of an abnormal GER documented by distal esophageal pH monitoring is estimated to be 8% in an unselected, asymptomatic sample of infants [4]. In our current study, which included only symptomatic infants who qualified for a 24-hour EPM study, 31.8% of patients had pathologic reflux. Reference values for age-related normal values from EPM in asymptomatic infants have been published from Europe [3,4]. Control data in asymptomatic infants is not available from the US as it would be difficult to perform such studies in North America due to ethical reasons.

In our definition of the GERD group, we used a reflux index of 5% as a cut off value similar to our previous studies [15]. Despite it being a different cutoff value as compared to the NASPGHN cutoff of 12% in the 1^st ^year of life, we feel that a 5% cutoff is more applicable to our patient population and our diagnostic equipment. This different cutoff percentage may result in a higher incidence of GERD in our study as compared to other studies; however previous studies were performed on asymptomatic kids in contrast to our symptomatic population [4]. The currently recommended normal values for EPM parameters are thought to be based on limited data from studies done on healthy infants with parameters not normally distributed [4], or studies with controls older than our study group [16]. There is a need for more normal data before EPM results can be confidently interpreted [17]. Data also depend on technical hardware such as recording devices and electrodes together with such patient characteristics as age, position, activity, and medication [18]. We recommend that even a lower cutoff value of reflux index for diagnosing GERD in infancy is needed to improve its sensitivity and specificity as a criteria of diagnosis [18]

Gender has been reported to play a role in reflux in healthy and symptomatic adults [5-7]. However, gender related values in infancy have not been evaluated previously. Richter et al [5] have shown that men tend to have more physiologic reflux than women in all EPM parameters from data on 110 healthy adults from three different centers. Fass [6] and associates reported that normal males had more variability and higher parameters in comparison to females for all values on pH monitoring. Ter [7] and coworkers assessed the same pH monitoring criteria in 353 symptomatic adults. Men had significantly more reflux and significantly higher values for all reflux parameters. In contrast, Shoenut et al [19] reported that the severity of reflux was not significantly different in adults between the two genders in referred symptomatic patients. It has been proposed that gender differences in parietal cell mass may account for this observation in adults [20]. Stomachs of men have more parietal cells and thus secrete more acid than women [20]. Based on these observations, it has been suggested that different gender-specific criteria be used in evaluating pH-monitoring results in adults. However, such size related differences between genders are unlikely to manifest in infancy.

There have been very limited data on gender differences in EPM parameters in infancy regarding both severity and prevalence in physiologic or pathologic reflux groups. A slight male preponderance for GER has been observed in pediatric studies. In asymptomatic infants it was 1.27:1 [21] and in a study of symptomatic infants and children it was 1.3:1 [22]. These figures are similar to our incidence of 1.3:1 for male: female. This incidence was present in both control and GERD groups as well as by ethnicity. Although our population is different from European infant data in that totally asymptomatic infants were screened for sudden infant death syndrome by pH monitoring and polysomnography by them [21]. Our patients are similar to data from Shepherd et al [22] in being symptomatic but their patients were older than our cohort.

Several studies in adults have shown that Caucasians have a higher frequency of symptoms, incidence, and complications of GERD (ulcers, strictures, or Barrett's esophagus) as compared to African Americans and Asians [23-26]. Caucasian ethnicity was also shown to be positively related to treatment satisfaction in adults with GERD [27]. A recent study in Thai infants also suggested that these infants had earlier resolution of regurgitation in comparison to their Western cohort [28]. Our present study showed a higher proportion of Caucasian infants having abnormal EPM parameters i.e. with GERD compared to AA infants. This suggests that racial differences in the incidence of GERD as previously reported in adults may also be present in infants [23-26]. This is a particularly valid observation as overall, majority of infants in our referral population are AA. We have observed this higher prevalence of GERD in Caucasians in another study as well [29]. Our data makes a strong case for racially associated genetic predisposition for GERD.

Dietary factors have been incriminated as one of the possible etiologies for increased incidence of GERD. However, most infants are usually on similar feedings regardless of ethnicity. Ostakul et al also did not observe any association of the prevalence of reflux regurgitation with the type of feeding i.e. breast milk vs. formula among Thai infants [28].

A lower concentration of gastric juice hydrogen ion concentration was reported in basal state and after pentagastrin stimulation in adult AA as compared to Caucasians [30] which may explain ethnic predisposition for Caucasians in the etiology of GERD. Although unlikely in infancy, the role of these factors in the etiology of racial differences in infants remains to be determined. The higher trend of EPM parameters in Caucasian females in comparison to all other groups is another interesting observation in our study. This finding has not been previously reported in adults or in the pediatric population. A significant association between the body mass and symptoms of reflux has been reported in postmenopausal women with estrogens being implicated as etiology of GERD, however, we cannot speculate on such a hormonal factor in infancy [31]. Our Caucasian females were significantly older (5 months versus 3.5 months) as compared to the Caucasian males (p = 0.03) in the GERD group. Although GER symptoms peak at 4 months [32], such a disposition for higher reflux parameters has not been previously reported. Since the etiology of such racial differences in unclear, it is important to conduct further studies to better understand the racial differences and to overcome any racial disparities that may result from overlooking such differences [33].

The main limitation of our study is that it was a retrospective study which made it difficult to quantify the severity of symptoms on referral. It should also be noted that our study was conducted in an urban tertiary care center setting with the majority of patients being of African American origin. The ethnic distribution of patients and time of referral to such a center may play a role in determining the incidence of GERD.

## Conclusion

Different set of control data to compare the EPM results between the two genders do not appear warranted in infants younger than 1 year of age. Higher prevalence of GERD in Caucasian infants should be considered during evaluation for symptoms suggestive of reflux. Further prospective studies are needed in infants to corroborate our observations of the higher reflux parameters in Caucasian females.

## Competing interests

The author(s) declare that they have no competing interests.

## Authors' contributions

DN was responsible for collecting and interpreting the data. DN wrote the final manuscript as well as early versions. VT was the project leader for this manuscript, edited the study design, interpreted the data and edited the final manuscript. VT gave the final approval of the version to be published. RT did the statistical analysis and interpreted the data. All authors read and approved the final manuscript.

## Pre-publication history

The pre-publication history for this paper can be accessed here:


